# Evaluating the Noninferiority of a New Photodynamic Therapy (Flexitheralight) Compared With Conventional Treatment for Actinic Keratosis: Protocol for a Phase 2 Study

**DOI:** 10.2196/11530

**Published:** 2019-04-26

**Authors:** Fabienne Lecomte, Anne Sophie Vignion-Dewalle, Claire Vicentini, Elise Thecua, Pascal Deleporte, Alain Duhamel, Serge Mordon, Laurent Mortier

**Affiliations:** 1 U1189 - Image Assisted Laser Therapies for Oncology, Inserm Centre Hospitalier et Universitaire de Lille Université de Lille Lille France; 2 Department of Dermatology Centre Hospitalier et Universitaire de Lille Université de Lille Lille France; 3 EA 2694 - Santé Publique: épidémiologie et Qualité des Soins, Unité de Biostatistiques Centre Hospitalier et Universitaire de Lille Université de Lille Lille France

**Keywords:** photodynamic therapy, actinic keratosis, light-emitting fabrics, Aktilite CL 128 (Galderma Laboratories)

## Abstract

**Background:**

Actinic keratosis (AK) is characterized by preinvasive, cancerous lesions on sun-exposed skin that negatively affect patient quality of life and may progress to invasive squamous cell carcinoma (SCC). If untreated, AK may either regress or progress to SCC, with significant morbidity and possible lethal outcomes. The most commonly used treatments for AK are cryotherapy, topical chemotherapy and, more recently, photodynamic therapy (PDT). This clinical study is part of a project that aims to create specific light-emitting fabrics (LEFs) that strongly improve the efficiency and reliability of PDT as a treatment for AK.

**Objective:**

This study aims to compare the efficacy and tolerability of a new PDT protocol involving the Flexitheralight device (N-PDT) with the classical protocol involving the Aktilite CL 128 device (C-PDT; Galderma Laboratories) for the treatment of AK. All participants receive both protocols. The primary objective of this study is to compare the lesion response rate after 3 months of N-PDT with C-PDT. Secondary objectives are evaluations of pain and local tolerance during treatment, clinical evolution of the subject's skin, and evaluations of patient quality of life and satisfaction.

**Methods:**

The study is a split-face, intraindividual comparison of two PDT protocols. The total number of patients recruited was 42. Patients were exposed to a continuous red light with the Aktilite CL 128 device on one side of the face and to fractionated red illumination with the new device, Flexitheralight, on the other side of the face. Males or females over the age of 18 years with a clinical diagnosis of at least 10 previously untreated, nonpigmented, nonhyperkeratotic grade I and II AK lesions of the forehead and/or scalp were included and were recruited from the Department of Dermatology of the Centre Hospitalier Universitaire de Lille. The patients came to the investigational center for one treatment session (day 1), and they were followed up after 7 days, 3 months and 6 months. A second treatment session was performed on day 111 in cases in which an incomplete response was observed at the 3-month follow-up. Data will be analyzed using SAS software version 9.4 (SAS Institute Inc). Continuous variables will be reported as means and standard deviations, and categorical variables will be reported as frequencies and percentages. The Shapiro-Wilk test will be used to assess the normality of the distribution.

**Results:**

The clinical investigation was performed by July 2018. Data analysis was performed at the end of 2018, and results are expected to be published in early 2019.

**Conclusions:**

This phase II clinical trial aims to evaluate the noninferior efficacy and superior tolerability of N-PDT compared to that of C-PDT. If N-PDT is both efficacious and tolerable, N-PDT could become the treatment of choice for AK due to its ease of implementation in hospitals.

**Trial Registration:**

ClinicalTrials.gov NCT03076918; https://clinicaltrials.gov/ct2/show/NCT03076918 (archived by WebCite at http://www.webcitation.org/771KA0SSK)

**International Registered Report Identifier (IRRID):**

DERR1-10.2196/11530

## Introduction

Actinic keratosis (AK) is characterized by common, preinvasive, cancerous lesions in sun-exposed skin [1-4] that negatively affect the quality of life in patients and may progress to invasive squamous cell carcinoma (SCC) [5]. AK usually develops on areas that are frequently exposed to the sun (eg, face, ears, scalp, neck, forearms, backs of hands). Patients with AK often express embarrassment, worry, and irritation related to the change in appearance of their skin and the unsightly nature of the lesions [6]. In addition causing emotional strain, AK lesions can be painful and easily traumatized, causing bleeding [5,7-9]. If untreated, AK may either regress or progress to SCC, with significant morbidity and possible lethal outcomes [10]. The malignant potential and impossibility of predicting which AK lesions will evolve into SCC have led to the common consensus that AK lesions must be treated [11]. The most commonly used treatments for AK are cryotherapy, topical chemotherapy, and, more recently, photodynamic therapy (PDT) [2,12-16].

PDT is based on the activation of light-sensitive molecules (photosensitizers) that are preferentially localized in the diseased tissues, resulting in the formation of reactive oxygen species and subsequently tissue injury and cell death; 5-aminolevulinic (ALA) and its ester, methyl aminolevulinate (MAL), are both photosensitizer precursors that are most often used for topical PDT. After being topically applied to the skin, these photosensitizer precursors are endogenously converted by the heme biosynthetic pathway into the photosensitizer protoporphyrin IX (PpIX) and other intermediate photosensitizing porphyrins [17]. As abnormal cells accumulate substantially higher levels of PpIX than normal cells [18], the subsequent illumination leads to their selective destruction. PDT with MAL has been shown to be an attractive treatment modality for AK because it enables the treatment of large areas with a high response rate and an excellent cosmetic outcome [19-22].

Classical PDT (C-PDT) is already used, but it involves rigid, planar light source devices (like Aktilite C128, Galderma Laboratories) that do not allow the homogeneous illumination of convex surfaces such as the scalp. Therefore, the dermatologist does not know the actual light dose that is delivered during C-PDT, and some lesions may be undertreated. This limitation could explain some treatment failures [23]. Moreover, C-PDT is only available in specialized environments (hospitals and clinics) and has not been sufficiently developed and widely used.

This clinical study is part of a project that aims to create specific light-emitting fabrics (LEFs) that improve the efficiency and reliability of PDT [24] as a treatment for AK. Flexitheralight is a new device for PDT treatment (N-PDT; U1189, Inserm) that appears to be perfectly adapted for treating skin zones because of its homogeneity, low weight, flexibility, optimal conformability, and low cost. Moreover, the Flexitheralight device can be used at home, following the diagnosis and treatment definition by specialists.

## Methods

### Trial Design

The trial was a proof-of-concept study and was a comparative (split-face and intraindividual comparison), randomized, open-label, single-center evaluation of the noninferiority of N-PDT compared with C-PDT.

### Setting

The study was conducted at the Lille University Hospital in the Department of Dermatology over a period of 24 months until the end of 2017. Forty-two patients were included and were followed for 6 months.

### Device

**Figure 1:**
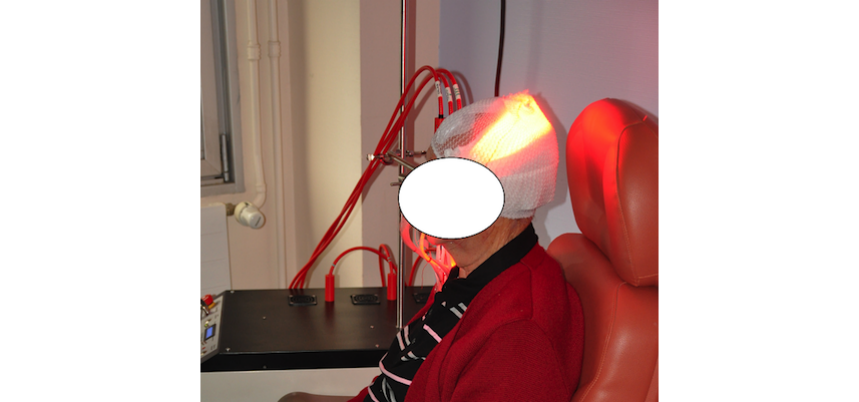
Flexitheralight device.

Flexitheralight is a new illumination device consisting of LEFs connected to a laser source (Figure 1). For fractionated illumination, 3 juxtaposed LEFs, each 20 cm x 5 cm, are positioned on the patient’s head.

Each LEF is connected to a 635 nm laser source, which is tuned to deliver an irradiance of 12.3 mW/cm^2^. This irradiance is controlled by a PD300 photodiode sensor connected to a StarBright laser power meter (both Ophir Optronics Solutions Ltd).

**Figure 2:**
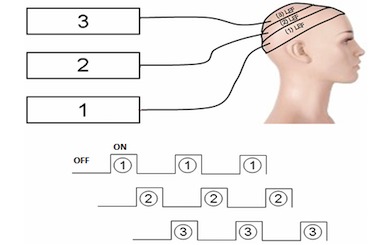
Fractionated illumination with LEF.

The 3 LEFs are activated sequentially as follows (Figure 2): on for 60 seconds and off for 120 seconds, with the sequence being repeated 50 times. When using these parameters, the total fluence is 37 J/cm^2^ for an illumination time of 2 hours and 30 minutes.

### Participants

To be eligible for the study, patients had to fulfill all the inclusion criteria described in Textbox 1 below. If they had only one of the noninclusion criteria, they were excluded from the study.

Information about the trial was provided to the patients, both orally and in a written format. Written informed consent was obtained from patients at the screening visit before entering the study.

The tolerability of the device was assessed on the first five patients. The study would have been completely interrupted if at least one patient had pain rated at 5 or higher out of 10 in the N-PDT area as measured by the pain assessment scale or at least one serious adverse event related to N-PDT occurred.

**Selection criteria.**

Inclusion criteria:

- Males or females over the age of 18 years
- Clinical diagnosis of at least 10 previously untreated, nonpigmented, nonhyperkeratotic, grade I and II actinic keratosis (AK) lesions of the forehead and/or scalp (according to Olsen et al [25])
- Other therapies are not unacceptable or considered medically less appropriate
- Symmetrical repartition of AK in terms of number and severity of lesions on both areas of the forehead and/or scalp. The axis of symmetry between the two areas is defined by the investigator according to the distribution of lesions
- AK is diagnosed upon a clinical evaluation (ie, visual inspection and palpation) performed by the investigator
- No treatment of AK received in the previous 30 days
- The two areas to be treated should not be coalescing. A minimum distance of 10 mm between the lesions located on the 2 symmetrical areas is required. A minimum distance of 2 mm between the lesions on the same area is required
- A minimum of 5 lesions and a maximum of 7 lesions with similar dimensions at both symmetrical areas are included. If the number of lesions is more than 7, only 7 lesions in each area are considered

Noninclusion criteria:

- Patients with porphyria
- Patients who are immunosuppressed for idiopathic, disease-specific, or therapeutic reasons
- Use of topical corticosteroids on the lesioned areas within 2 weeks before photodynamic therapy (PDT)
- Patients receiving local treatment (including cryotherapy, curettage-electrocoagulation, or any PDT treatment) of the face/scalp area within the last 30 days
- Patients receiving topical treatment (including imiquimod, fluorouracil, diclofenac, or ingenol mebutate) of the face/scalp area within the last 3 months
- Use of topical retinoids, alpha hydroxy acids, urea, or systemic retinoids or chemotherapy or immunotherapy within the 4 last weeks
- Pigmented AK lesions
- Known allergy to ester methyl aminolevulinate or similar PDT compound or excipients of the cream including arachis, peanut, or soya oil

- Participation in other clinical studies either currently or within the last 30 days
- Female subjects must meet one of the following criteria:
  - Nonchildbearing potential: postmenopausal or have a confirmed clinical history of sterility (eg, the subject does not have an uterus)
  - Childbearing potential: confirmed negative urine pregnancy test or blood analysis prior to study treatment to exclude pregnancy
- Any condition that may be associated with a risk of poor protocol compliance
- Patients currently receiving regular ultraviolet radiation therapy

### Study Objectives and Outcomes

The primary objective is the comparison of the lesion response rate 3 months after either N-PDT or C-PDT. Key secondary objectives are treatment tolerability, complete response rate after 6 months, cosmetic results, patient quality of life, and satisfaction (Table 1).

### Sample Size

The study is designed to have a statistical power of 80% with a one-sided alpha level of .025 to determine noninferiority in terms of a complete lesion response rate 3 months after N-PDT compared with C-PDT. Assuming a complete lesion response rate of 75% in both areas, an intrapatient correlation in both lesions and areas, and a noninferiority margin of 10%, the number of required lesions per area is 245. This value corresponds to 42 patients, assuming 12 lesions per patient (6 lesions per patient per area).

### Allocation and Randomization

Patients who met all of the eligibility criteria were included in the study by central randomization. The randomization schedule was generated by a statistician using the PROC PLAN procedure in SAS statistical software (SAS Institute Inc) with a 1:1 allocation ratio and a block size of 6. The allocation was concealed using sequentially numbered, opaque, sealed envelopes that were opened sequentially by the investigator at the beginning of the treatment.

### Implementation and Blinding

The study was not blinded, and patients and investigators knew the procedure allocation. Efficacy and tolerability were evaluated by investigators who knew the type of treatment assigned to each area. Data will also be analyzed without blinding.

**Table 1:** Criteria for objectives evaluation.

Outcome and description		Visit				
		1^a^	2^b^	3^c^	3bis^d^	4^e^
**Complete response rate**						
	Total disappearance of each lesion			x		x
	Number of patients presenting a 75% lesion reduction rate			x		x
**Tolerability**						
	Evaluation of pain (visual analogic scale)	x			x	
	Local tolerance (adverse event, serious adverse event, concomitant treatments)	x	x		x	
**Cosmetic results**						
	Clinical assessment of the subject’s skin aspect (excellent, good, fair, or poor)	x		x		x
**Quality of life and satisfaction**						
	DLQI^f^ and satisfaction questionnaire	x	x	x	x	x

^a^Visit 1: day 1.

^b^Visit 2: day 7.

^c^Visit 3: at 3 months.

^d^Visit 3bis: day 111.

^e^Visit 4: at 6 months.

^f^DLQI: Dermatology Life Quality Index.

**Figure 3:**
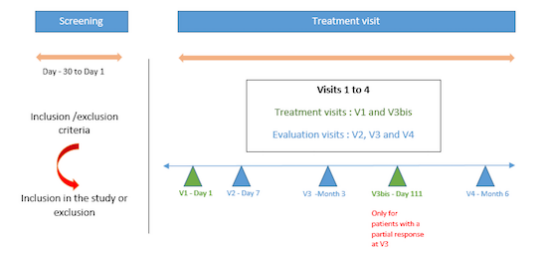
Schematic of the study procedure. V: visit.

### Interventions

As shown in Figure 3, after screening, patients who met all the inclusion criteria and none of the exclusion criteria were randomized and invited to come to the investigation site for 4 visits: day 1, day 7, month 3, and month 6. If an incomplete clinical response was observed at month 3, patients were retreated with PDT during visit 3bis on day 111.

#### Initial Visit: Preparation and Treatment of Lesions

##### Selection of Treatment Areas

**Figure 4:**
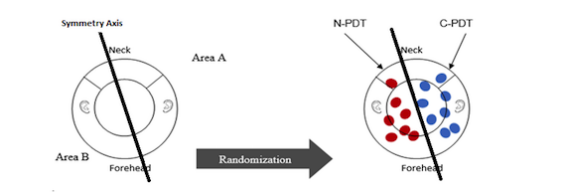
Schematic of the randomization process for area A and area B. C-PDT: Aktilite CL 128 device; N-PDT: Flexitheralight device.

Each subject’s skin aspect was evaluated, and the two areas were treated according to the study protocol and randomization design. Randomization was performed after the definition of the axis of symmetry to avoid selection errors (Figure 4).

The global area of the scalp and front of the face was divided into two symmetrical areas (area A and area B) containing the same number and same grades of AK lesions. The areas to be treated were localized between the eyebrows and the neck. Included AK lesions were located, counted, graded, and photographed.

For each patient, *n* lesions in area A were treated with one technique (N-PDT or C-PDT) and *n* lesions in area B were treated with the other technique (C-PDT or N-PDT) (5≤*n*≤7).

Before applying MAL, the areas were prepared by removing the crusts with a small curette and gently scraping the surface of the lesions to roughen the surface.

Pain in the two treated areas was scored by the patient after treatment: first for the N-PDT area and then for the C-PDT area.

Patients complete a quality of life questionnaire (Dermatology Life Quality Index [DLQI]) and a satisfaction questionnaire at the end of the procedure.

The total duration of the treatment procedure (treatment of areas A and B) was approximately 3 hours and 20 minutes.

##### Area A: Classical Photodynamic Therapy

**Figure 5:**
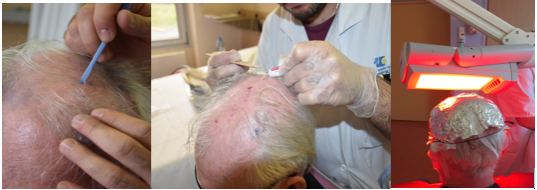
Illustration of the classical photodynamic therapy treatment procedure.

MAL was applied (approximately 1 mm thick) with a spatula on the selected lesions and over an area of 5 to 10 mm of normal skin surrounding the lesions. The treated area was covered with an occlusive (Tegaderm, 3M) and light-proof (aluminium foil) dressing for 3 hours. Afterward, the dressing was removed, the area was cleaned with a saline solution, and the skin was then immediately exposed to a continuous red light spectrum delivered by an Aktilite CL 128 device (Galderma Laboratories) (570 to 670 nm) for 10 minutes for a total light dose of 37 J/cm^2^ (Figure 5).

##### Area B: New Photodynamic Therapy

**Figure 6:**
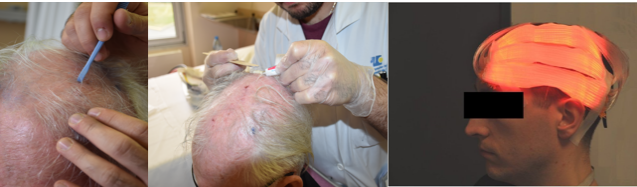
Illustration of the new photodynamic therapy treatment procedure.

MAL was applied as described for the area A treatment, and the area was covered with an occlusive and transparent dressing (Tegaderm, 3M) for 30 minutes whereas both a transparent occlusive dressing and a light-proof dressing (aluminium foil) was applied over the area randomized to receive C-PDT. Afterward, the dressing was retained, and irradiation was applied with the Flexitheralight device (635 nm) for 2 hours and 30 minutes. A total light dose of 37 J/cm^2^ was administered (Figure 6). After the end of the illumination, area B was protected with aluminium foil.

#### Follow-Up and Retreatment Visits

Visit 2 occurred 7 days after treatment to evaluate the tolerability and adverse effects of the treatments. Patients completed the DLQI and satisfaction questionnaires. Photographs of the treated areas were captured under standardized conditions.

Visit 3 occurred 3 months after treatment. The investigator evaluated the response to treatment by comparing the lesions between the current visit and the first visit (by referring to paper tracings and photographs taken during the first visit). If some of the treated AK lesions remained, a new visit was scheduled within 3 weeks to treat the remaining lesions. The remaining lesions in each area were located, counted, and graded. Only the presence of lesions was considered and not any changes in their sizes. If a new lesion appeared, it was treated (by the same procedure), but it was not considered for the comparison of lesions between months 3 and 6. Photographs of the two treated areas were taken. Patients completed the DLQI and the satisfaction questionnaires, and all adverse events and concomitant medications were recorded. Patients for whom the AK lesions had completely disappeared were invited to participate in an assessment visit at month 6.

Visit 3bis was optional and scheduled only in cases where at least one AK lesion remained after the first treatment session and only if the investigator considered it necessary for the subject to be treated again with PDT. The same treatment was applied as in visit 1.

Visit 4 occurred 6 months after the initial treatment. The investigator evaluated the treatment response by comparing the lesions between the current visit and the first visit. Photographs of the two treated areas were taken. Patients completed the DLQI and the satisfaction questionnaires, and all adverse events and concomitant medications were recorded.

### Variables and Data Collection

Collected data consisted of demographic data, medical history reviews, previous radiotherapy histories, history of surgery and treatment for AK, definition of AK lesions (localization, number, grade, and photographs), and assessments of the subjects’ skin aspects.

For women of childbearing age, a urine pregnancy test was performed at screening or before the beginning of the treatment.

Several scales (pain, aesthetic aspect, and treatment tolerance) and questionnaires (DLQI and satisfaction) were used.

### Data Management

All medical observations were maintained in the patient’s file; the data to be analyzed in the study were reported on an electronic case report form according to Good Clinical Practices and the sponsor’s standard operating procedures. The data collection procedure was exhaustive and verified regularly by a clinical research associate according to the protocol. Any deviation from the protocol was noted, and the reason for the deviation was documented. Discrepancies in the data were brought to the attention of the clinical team and investigational site personnel in the form of a query. Resolutions to these issues are reflected in the database.

### Statistical Methods

Continuous variables will be reported as means and standard deviations, and categorical variables will be reported as frequencies and percentages. The Shapiro-Wilk test will be used to assess the normality of the distribution. This normality will also be evaluated graphically.

#### Analysis of Primary Objective

In this study, each patient could have several lesions. We considered the “patient” effect. Indeed, a correlation could exist between the outcome measures in a single patient (cluster effect). The complete response rate of lesions will be analyzed according to the treatment groups (N-PDT or C-PDT) using the generalized linear mixed model to consider the cluster effect with an adjustment for the period (by the area). The 95% confidence interval of the absolute difference in response rates between the two groups will be calculated (D=N-PDT – C-PDT). We will conclude noninferiority if the lower limit of this 95% confidence interval is greater than 10%. If noninferiority is confirmed, a superiority test will be performed.

#### Analysis of Secondary Objectives

The percentage of patients in each group with a reduction in the lesion number greater than 75% will be calculated and compared using a generalized linear mixed model. The aforementioned method will be used for comparisons of the other qualitative variables between the two groups (N-PDT or C-PDT). For continuous variables, we will use the linear mixed model. The pain levels reported at the end of each treatment will be compared using a linear mixed model, with patients as the random effects (the significance level will be set to .05). All statistical analyses will be performed using SAS software version 9.4 (SAS Institute Inc).

### Ethical Approval

This study was performed in accordance with the ethical principles of the Declaration of Helsinki (2008) and the International Conference on Harmonisation–Good Clinical Practices and in compliance with Article L. 1121-4 of the French Public Health Code. The study design was reviewed and approved by the French National Agency for the Safety of Medicines and Health Products (authorization number 2013-A01096-39) and the French Ethics Committee (authorization number CPP-03/051/2013).

## Results

**Figure 7:**
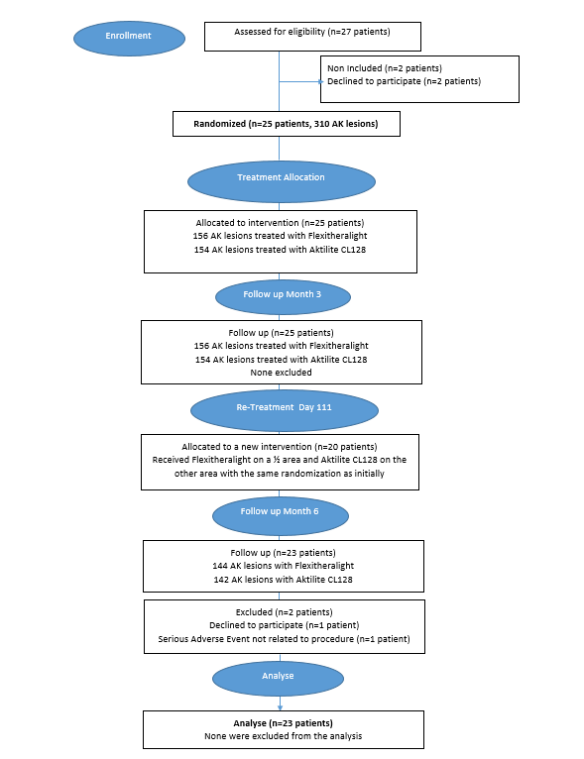
Study flowchart. AK: actinic keratosis.

Figure 7 shows the evolution of the number of subjects included, followed, and considered in the statistical analysis. Enrollment is closed. A total of 27 patients were recruited and followed instead of the planned 42 subjects due to the early termination of the Flexitheralight study, resulting from the launch of the competing Phos-Istos European study. Of the 27 patients, 23 completed all visits of the study.

The clinical investigation was performed by July 2018. Data analysis was performed at the end of 2018, and results are expected to be published in early 2019.

## Discussion

As part of the primary objective, we hope to demonstrate that N-PDT is not inferior to C-PDT in terms of the lesion response rate at month 3. As part of the secondary objectives, we seek to demonstrate that N-PDT is less painful and better tolerated than C-PDT as a treatment for AK.

The adverse effects associated with C-PDT are usually a local reaction at the treatment site that is attributable to the toxic effects of PDT (phototoxicity) or to the preparation of the lesion. The most common symptoms are pain and discomfort, which are described as burning and stinging sensations, erythema, and encrusting sensations of skin pain. Usually, the symptoms begin with or immediately after illumination, last for a few hours, and disappear on the day of treatment.

The possible risks related to N-PDT have been analyzed. Based on the results from this analysis, the Flexitheralight device has been classified as an exempt risk group, according to International Electrotechnical Commission 60601-2-57/2012.

Regarding the irradiance, the objective was to deliver 12.3 mW/cm^2^, lower than the 75 mW/cm^2^ irradiance delivered by the Aktilite CL 128 device or the 22 mW/cm^2^ delivered by sunlight at midday in the summer in Munich. The expected benefit for patients included in the study is a reduction of pain experienced during treatment, increasing comfort. Indeed, illumination during C-PDT is intensively administered for a short period of time, which is known to increase pain [26].

In addition to the impact on pain, the flexibility of the Flexitheralight device enables a homogeneous illumination, which should yield better efficiency. Moreover, N-PDT could be performed in all weather conditions, in any geographic location, year round, and could therefore become the treatment of choice for AK.

## Abbreviations

AK: actinic keratosis
ALA: aminolevulinic acid
C-PDT: classical photodynamic therapy
DLQI: Dermatology Life Quality Index
LEF: light-emitting fabric
MAL: ester methyl aminolevulinate
N-PDT: new photodynamic therapy
PDT: photodynamic therapy
PpIX: protoporphyrin IX
SCC: squamous cell carcinoma
